# A novel machine learning model of smartphone-based 1-minute sit-to-stand test for prediction of six-minute walk test distance in patients with COPD

**DOI:** 10.1016/j.clinsp.2026.100923

**Published:** 2026-04-03

**Authors:** Simin Xie, Xiao Ge, Lin Huang, Wenyu Zhu, Yue Yang, Min Zhou, Jieming Qu, Yi Guo

**Affiliations:** a Department of Pulmonary and Critical Care Medicine, Ruijin Hospital, Shanghai Jiao Tong University School of Medicine, Shanghai, China; b Institute of Respiratory Diseases, Shanghai Jiao Tong University School of Medicine, Shanghai, China; c Shanghai Key Laboratory of Emergency Prevention, Diagnosis and Treatment of Respiratory Infectious Diseases, Shanghai, China; d Luca Healthcare (Shanghai) Co., Ltd, China

**Keywords:** Chronic Obstructive Pulmonary Disease (COPD), 1-Minute Sit To Stand Test (1MSTST), 6-Minute Walk Test (6MWT), Machine Learning Model, Smartphone

## Abstract

**Background:**

Regular assessment of exercise tolerance is essential for managing COPD, nevertheless, the standard 6-Minute Walk Test (6MWT) is difficult to perform outside clinical settings. This study aimed to develop and validate a smartphone-based digital 1-Minute Sit-to-Stand Test (1MSTST) to estimate 6-Minute Walking Distance (6MWD) by integrating data from the phone's Inertial Motion Unit (IMU) with advanced machine learning algorithms, offering a convenient alternative for remote functional assessment.

**Methods:**

The enrolled COPD patients completed the smartphone-based digital 1MSTST and 6MWT with a minimum 15-minute rest period between the two tests. Accelerometer and gyroscope data were recorded by a smartphone throughout the 1MSTST. Systolic and Diastolic Blood Pressure (SBP, DBP), Heart Rate (HR) and Pulse Oxygen Saturation (SpO_2_) were measured before and after the 1MSTST and 6MWT. The authors used a smartphone-based dataset of 66 subjects and algorithms such as Extreme Gradient Boosting (XGBoost), Light Gradient Boosting Machine (LightGBM), Support Vector Machine (SVM), Linear Regression (LR) and Random Forest (RF) to estimate the prediction of digital 1MSTST to 6MWD. The correlation between the features extracted from digital 1MSTST and the 6MWD was analyzed.

**Results:**

A total of 66 patients with stable COPD were enrolled to build the predictive model for 6MWD. The change of HR and SBP after 1MSTST was higher than that of 6MWT (paired *t*-test, ΔHR: *p* < 0.0001, ΔSBP: *p* < 0.0001) with no significant difference in the change of DBP and SpO_2_ (paired *t*-test, ΔDBP: *p* = 0.974, ΔSpO_2_: *p* = 0.072). Pearson correlation analysis identified the top seven features that were strongly correlated with 6MWD. The RF machine learning model had the best performance to predict the 6MWD (R-square = 0.86, MAE = 24.3). Bland-Altman analysis indicates that the RF model provides a bias of -0.61 ± 31.04 and that the limits of agreement (95%, 1.96 SD) range from -60.22 to 61.45.

**Conclusions:**

The smartphone-based digital 1MSTST, combined with machine learning, can accurately estimate 6MWD. The significance of this study lies in proposing a novel assessment paradigm that may serve as a practical tool for remote monitoring of exercise capacity in COPD management.

## Highlights

• The 1-minute sit-to-stand test and 6-minute walk test are key tools for evaluating exercise capacity in COPD patients.
• A smartphone-based 1-minute sit-to-stand test predicts the 6-minute walk distance.
• Combines inertial motion unit sensor data with machine learning for accurate estimation.
• Enables remote COPD management using smartphone sensors and advanced algorithms.

## Introduction

Chronic Obstructive Pulmonary Disease (COPD) is a chronic airway disease characterized by persistent respiratory symptoms and airflow limitation, with high morbidity and mortality rates over the years, resulting in a huge socioeconomic burden.^1^ The assessment of exercise capacity in patients with COPD is a valuable tool for evaluating the severity of the disease, monitoring its progression and guiding treatment decisions.

There are different methods to assess exercise capacity in COPD, such as field tests, laboratory tests, and self-reported questionnaires. Commonly used field tests, with high accessibility and objectivity, are the 6-Minute Walking Test (6MWT), the incremental shuttle walk test and the endurance shuttle walk test.^2^ Among these, the 6MWT is considered a widely utilized, validated and reliable test to evaluate the cardiopulmonary and musculoskeletal function in COPD,^3^ but the requirement of 30-meter corridor and falling risk often limits its use in private homes or out-of-hospital settings. More recently, new tests have been developed to facilitate exercise capacity in COPD, and in this context, 1-Minute Sit-to-Stand Test (1MSTST) has been increasingly studied as an alternative because it is safer and requires limited space.^4^ 1MSTST is valid and reliable for functional assessment in adults and children, proved to have a significant correlation to 6-Minute Walking Distance (6MWD).^5^, ^6^, ^7^, ^8^ 1MSTST has shown predictive validity as a strong and independent predictor of mortality and health-related quality of life in COPD patients.^9^

In recent years, with the development of smart technology, based on the traditional 1MSTST mentioned above, the rapid increase in smart devices and algorithm availability allows the exploration of digital 1MSTST. Current studies utilize different technologies, such as Inertial Motion Unit (IMU) or camera, focusing on unsupervised or remote improvements and simplifying the test process. 10, 11, 12 However, most studies assess the performance empirically based on the number of repetitions during 1MSTST, disregarding the massive, valuable clues underlying the digital manners.^13^ To the best of the authors' knowledge, no study has yet systematically validated a smartphone-based 1MSTST model capable of accurately estimating 6MWD in COPD patients. Addressing this gap could enable at-home monitoring of exercise tolerance using widely available mobile devices. Therefore, the present study is dedicated to developing a smartphone-based 1MSTST tool to predict 6MWD, presenting a new paradigm that fully exploits the combination of IMU data and machine learning models, also for the assessment of cardiorespiratory exercise function in COPD patients.

## Methods

### Study participants

Sixty-six patients with stable COPD were recruited for this study, according to the Global Initiative for Chronic Obstructive Lung Disease (GOLD) criteria. Inclusion criteria included age ≥ 40 years, a diagnosis of COPD, along with the ability to perform the 6MWT and 1MSTST. Exclusion criteria were acute exacerbations of respiratory symptoms within the past 2 weeks or significant comorbid cardiovascular, neurological, musculoskeletal, immunological, or infectious diseases. Medical records were used to collect data on baseline demographic information and clinical characteristics, including age, sex, Body Mass Index (BMI), and smoking history, etc. The Ethics Committee of Ruijin Hospital, Shanghai Jiao Tong University School of Medicine, approved this study (Approval n° 2023–199). All participants provided informed consent before enrollment. https://clinicaltrials.gov/ct2/show/NCT06082791. This study was reported in accordance with the CONSORT Statement rules for Clinical Trials.

### Spirometry

The pulmonary function tests of the enrolled patients were conducted by professional pulmonary function technicians in accordance with the pulmonary function test procedures on the MasterScreen PFT System pulmonary function tomography instrument (Jaeger, Germany). Before measurement, routine calibration was carried out to ensure the accuracy of the flow sensor and gas analyzer. Each test was repeated three times. The best result was recorded and compared it with the predicted value to calculate the percentage predicted value (%pred). All tests were conducted in accordance with the standardized operating procedures to ensure the accuracy and repeatability of the data.

### The smartphone-based digital 1MSTST

**Fig. 1:**
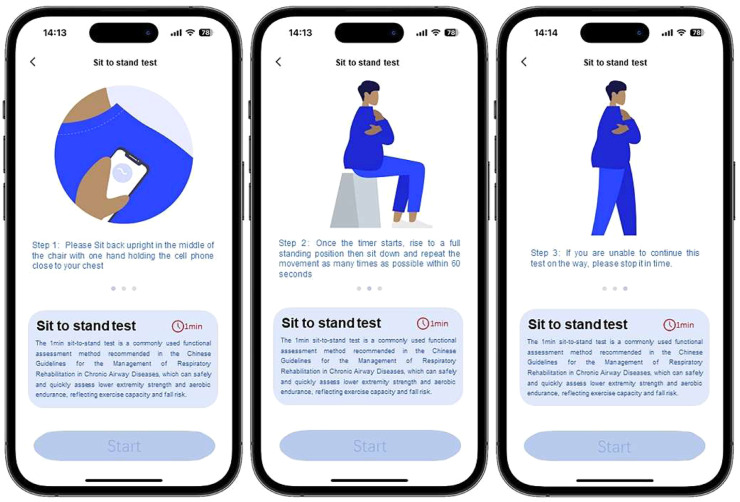
User interface of digital 1MSTST application.

The procedures of the digital 1MSTST followed the consensus recommendation.^14^ Unlike the traditional 1MSTST, the digital 1MSTST requires patients to hold a smartphone-based accelerometer and gyroscope. The digital 1MSTST was performed using a smartphone application built on the LucaPlex® platform and run-on an iPhone 12. The user interface was designed with an animated guide, introduction of 1MSTST, “Start Test” button (Fig. 1). The participants sat on an armless chair (height 45-50 cm) with the back straight, held the phone with either hand and crossed arms over the chest, with the phone screen facing inward and close to the chest. Participants clicked the “Start Test” button. After a five-second countdown, a voice announced “Start Test” to cue the start of testing. The patient sat upright in a chair, with legs hip-width apart and knees bent at a 90° angle, feet flat on the floor. The arms were crossed over the chest, without using hands or arms to assist the movement. Patients were instructed to repeat the standard motion of standing up and sitting down as many times as possible within 1-minute. After 45 seconds, the patient was reminded, “15-seconds remaining”.

### The 6-Min Walking Test (6MWT)

The 6MWT was performed following the international recommendation.^3^ Patients were asked to walk as far as possible in 6 minutes along a flat and marked 30 m corridor. The distance in metres was recorded. Participants were allowed to take breaks during the test.

All the patients completed the digital 1MSTST and 6MWT. A minimum 15-minute rest interval was provided between the two tests to avoid carry-over effects and ensure hemodynamic stabilization. Previous studies have used similar intervals to minimize fatigue when comparing exercise capacity tests in COPD patients.^5^^,^^22^ The changes of Systolic and Diastolic Blood Pressure (ΔSBP, ΔDBP), Heart Rate (ΔHR), and Pulse Oxygen Saturation (ΔSpO_2_) were measured at the beginning and the end of 1MSTST and 6MWT, respectively.

### Feature extraction

Accelerometer and gyroscope data were recorded throughout the digital 1MSTST at a 100 Hz sampling rate. The 3-axis accelerometer and gyroscope data were pre-processed with finite impulse response low-pass filtering and moving average filtering. Peaks and troughs were detected from the accelerometer signals and screened to identify sit-to-stand transitions. This algorithm achieved 96% accuracy in sit-to-stand repetitions with average absolute error < 0.1 s in determining sitting and standing timestamps. The raw data were then segmented and labeled with timestamps of the start and end of sit-stand transitions, and the feature metrics were subsequently analyzed.

Feature engineering involved creating interaction terms between relevant features to capture non-linear relationships and normalizing features to ensure equal contributions from variables with different scales.

For feature selection, features with high correlation or autocorrelation were removed to address multicollinearity and temporal dependencies. Recursive Feature Elimination (RFE) was then used to identify the most informative subset of features.

### Statistical analysis

Statistical analyses were conducted using R-4.3.3 statistical software. Descriptive data were reported as mean ± Standard Deviation (SD) or median and interquartile range, depending on the normality of the distribution. Qualitative variables were expressed as percentages. Paired *t*-tests were used to evaluate the differences in ΔHR, ΔSBP, ΔDBP and ΔSpO_2_ between the 6MWT and 1MSTST. The correlation between the results of the digital 1MSTST and 6MWD was calculated by Pearson's and Spearman's rank correlation coefficient. A p-value of <0.05 was considered significant.

### Predictive model development

Random Forest (RF), Linear Regression (LR), Support Vector Machine (SVM), Extreme Gradient Boosting (XGBoost), and Light Gradient Boosting Machine (LightGBM) were used to evaluate the efficacy of digital 1MSTST results to predict 6MWD. Five-fold cross-validation was employed to evaluate the performance of these models and prevent overfitting. Hyperparameter tuning was conducted within each fold to optimize model performance. The performance of the models was evaluated using two metrics: R-squared (R^2^) and Mean Absolute Error (MAE). A Bland-Altman analysis was performed to graphically examine the limits of agreement between the 6MWD predicted by 1MSTST and the actual 6MWD.

## Results

### Patient characteristics

Sixty-six COPD patients aged ≥ 40-years were included, including 61 males (92.4%). The demographics and clinical characteristics are described in Table 1. The mean age and BMI of the 66 subjects were 68.38 years and 22.85 kg/m^2^. The majority of subjects were in GOLD stages 2-3, with 31 patients (47%) in GOLD stage 2 and 26 patients (39.4%) in GOLD stage 3, respectively. The median of forced expiratory volume in 1s/prediction (FEV_1_%pre), the Forced Expiratory Volume in 1s/Forced Vital Capacity (FEV_1_/FVC) and the Diffusion Lung Capacity for Carbon Monoxide/prediction (DLCO%pre) was 50.00%, 54.55% and 63.80%, respectively.

**Table 1:** Characteristics of enrolled COPD patients.

Valuable	Value
Age (years)	68.38 ± 6.87
Sex male	61 (92.4%)
BMI (kg/m^2^)	22.85 ± 3.16
GOLD	
I	3 (4.5%)
II	31 (47%)
III	26 (39.4%)
IV	6 (9.1%)
CAT score	12.78±6.54
FEV_1_%pre	50.00 (38.95, 60.75)
FEV_1_/FVC	54.55 (46.62, 66.42)
DLCO%pre	63.80 (56.05,84.50)
6MWD (m)	417.5 (350, 450)
Digital 1MSTST (counts)	23 (18, 28)

Data are presented as mean ± SD, n (%) or median (interquartile range). BMI, Body Mass Index; CAT, COPD Assessment Test; FEV1%pre, forced expiratory volume in 1s/prediction; FEV1/FVC, forced expiratory volume in 1s/forced vital capacity; DLCO%pre, Diffusion Lung Capacity for Carbon monoxide/Prediction; 6MWD, 6-Minute Walking Distance; 1MSTST, 1-Minute Sit-to-Stand Test.

### Data of digital 1MSTST and 6MWT

**Fig. 2:**
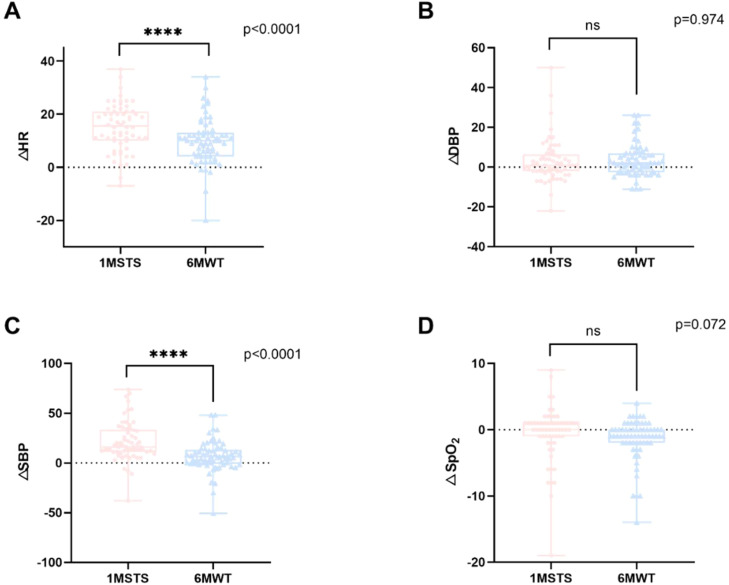
Comparison of vital sign changes between the 6MWT and 1MSTST. (A) The change in heart rate after the 6MWT or 1MSTST. (B) The change in diastolic blood pressure after the 6MWT or 1MSTST. (C) The change in systolic blood pressure after the 6MWT or 1MSTST. (D) The change in pulse oxygen saturation after the 6MWT or 1MSTST. ΔHR, change in heart rate; ΔDBP, change in Diastolic Blood Pressure; ΔSBP, change in Systolic Blood Pressure; ΔSpO_2_, change in Pulse Oxygen Saturation.

Regarding the physical capacity, the median number of repetitions in the 1MSTST was 23, and the median number of 6MWD was 417.5 m The changes in the vital signs of the participants after 1MSTST and 6MWT are shown in Fig. 2. Paired *t*-tests revealed significant differences in ΔHR (*p* < 0.0001) and ΔSBP (*p* < 0.0001) between the 6MWT and 1MSTST, while no significant differences were found in ΔDBP (*p* = 0.974) and ΔSpO_2_ (*p* = 0.072).

### Correlation analysis

The authors explored the relationship between 39 features extracted from digital 1MSTST and the 6MWD using Pearson's correlation coefficient. After calculating these coefficients, the authors sorted the features based on the absolute values of their correlations in descending order to identify the top 7 features that exhibit the strongest linear relationships with the 6MWD (Table 2). The correlation coefficients' direction (positive or negative) and magnitude indicated the strength of these relationships. Additionally, the authors assessed the statistical significance of these correlations to confirm the reliability and practical relevance of the findings.

**Table 2:** Interpretation of the most significant features.

Features	Definition	Interpretation
Walking distance (Distance)	The distance walked in 6MWT	Reflects the overall walking ability and endurance
Count of activities (Count)	The number of sit and stand actions in 1 min	Shows the frequency of posture changes
Mean sit-to-stand time (Mean)	The average time of a sit-to-stand transition	Offers insight into the speed and fluidity of movements
Distribution of Sitting (Down%)	Proportion of time spent in a seated position relative to the total time of sitting and standing activities	Patients who are prone to fatigue are more likely to have longer seated time
Sample Entropy (SampEn)	sample entropy(m,n,r) = $-\ln[\frac{B^{m+1}\left(r\right)}{B^{m}\left(r\right)}]$; where N is the length of the sample, m = 2 and r = 0.2*$SD_{x}$, $B^{m}\left(r\right)=\;\frac{1}{N-m+1}\sum_{i=1}^{N-m+1}B_{i}^{m}\left(r\right)$	Elucidating the power control and posture stability in a repeated movement task
Maximum Acceleration (Acc_max)	Maximum value of the modulus of acceleration	Unveils the extremes of force exertion
Coefficient of Variation in Gravity (G_cv)	Coefficient of variation in acceleration along the gravity axis	Provides a nuanced understanding of how we adapt and adjust to the pull of gravity
Variation in Body Angles (Trunk_ang)	Maximum forward trunk lean angle in the stand-up stage	Reflects the stability and muscle control ability during sit-to-stand

**Fig. 3:**
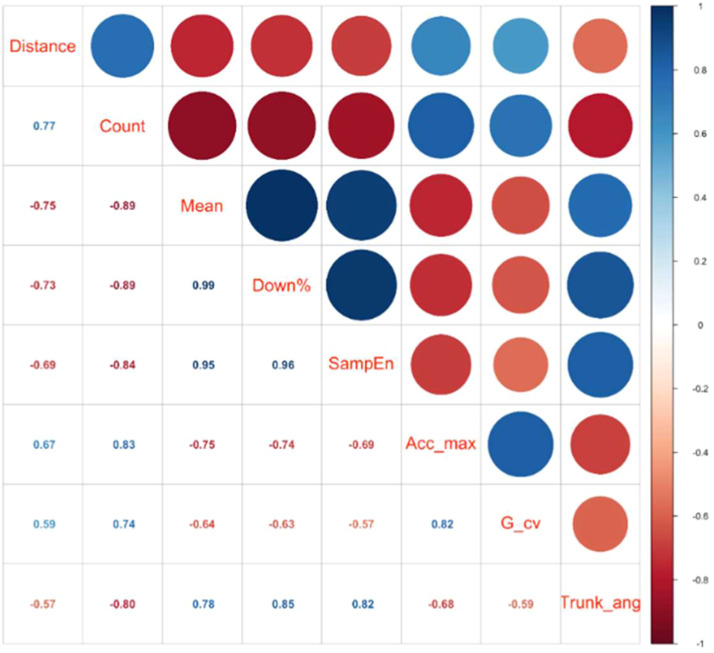
Correlation plots showing Pearson correlation values of 6MWD and digital 1MSTST features. Distance, 6-minute walk distance; Count, 1MSTST repetition counts; Mean, mean 1-min sit-to-stand duration; Down%, sitting time engaged in full sit-to-stand cycles; SampEn, Sample entropy of modulus of accelerometer; Acc_max, maximum accelerometer values across all 3-axes; G_cv, coefficient of variation in acceleration along the gravity axis; Trunk_ang, forward trunk angle during standing.

The 6MWD was positively correlated with digital 1MSTST repetition counts (*r* = 0.77, *p* < 0.01), the coefficient of variation in acceleration along the gravity axis (*r* = 0.59, *p* < 0.01), and the maximum accelerometer values across all 3-axes (*r* = 0.67, *p* < 0.01). In contrast, the 6MWD was negatively correlated with the sample entropy of the modulus of the accelerometer (*r* = −0.69, *p* < 0.01) and forward trunk angle during standing (*r* = −0.57, *p* < 0.01). Additional features demonstrated moderate correlation with the percentage of sitting time engaged in full sit-to-stand cycles (*r* = −0.73, *p* < 0.01) and the mean 1 min sit-to-stand duration (*r* = −0.75, *p* < 0.01) (Fig. 3). These features not only captured movement patterns and postural control during the 1MSTST, but also reflected differences in lower-limb strength, coordination, and endurance among patients.

### Predicted model of digital 1MSTST

**Fig. 4:**
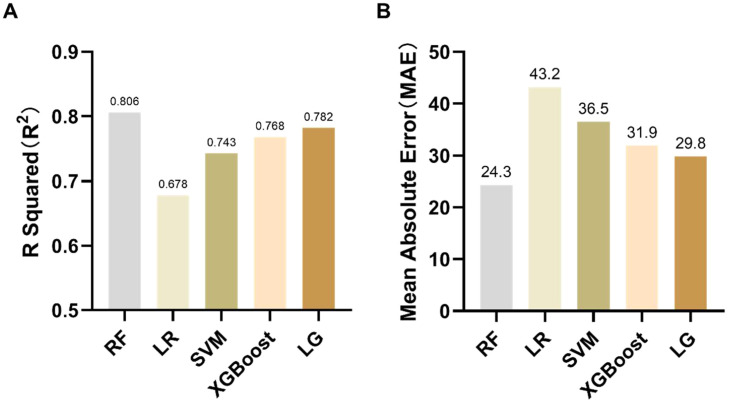
The performances of the models in terms of R squared (R^2^) and mean absolute error (MAE). RF, Random Forest model; LR, Linear Regression model; SVM, Support Vector Machine; LG, LightGBM model.

Building upon these key features, the authors further developed and compared multiple machine learning models, including RF, LR, SVM, XGBoost, and LightGBM, to evaluate the predictive capability of the digital 1MSTST for estimating 6MWD. This approach aimed to establish a link between signal-derived digital features and functional exercise capacity, thereby ensuring both physiological interpretability and predictive reliability of the proposed models. To evaluate the reliability of the models, R2 and MAE were used as common evaluation criteria. R2 quantifies the degree of alignment between the 6MWD predicted by the 1MSTST algorithm and the actual results of 6MWT. The random forest model achieved the highest R^2^-value (0.806), indicating its superior explanatory power for the target variable. However, the LR, SVM, XGBoost, and LightGBM models also achieved relatively high predictive performance, with R^2^ values of 0.678, 0.743, 0.768, and 0.782, respectively. (Fig. 4a). In addition, MAE quantifies the average absolute difference between the 1MSTST algorithm's predicted 6MWD and the actual 6MWT. The MAE values varied in the different models, ranging from 24.3 for the Random Forest model to 43.2 for the Linear Regression model. The Random Forest model also had the lowest MAE value, indicating that it made the most accurate predictions on average (Fig. 4b).

### Random forest model evaluation with test data

**Fig. 5:**
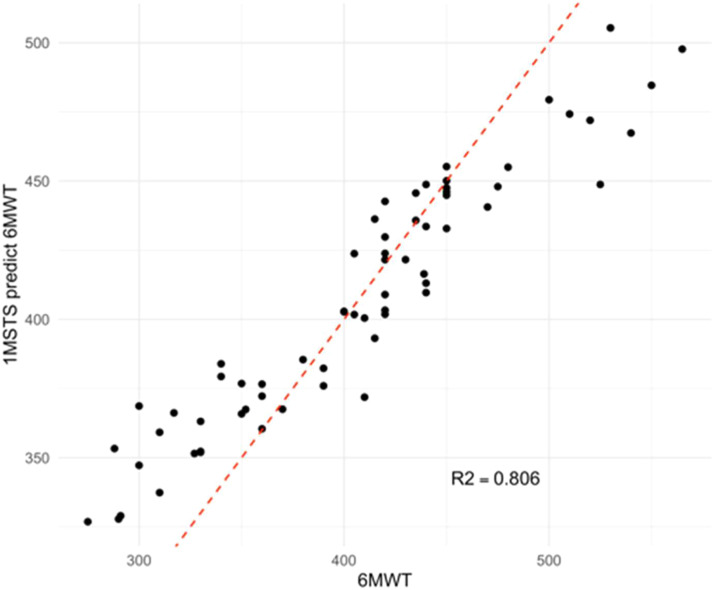
Fitted plot after Random Forest regression. The x-axis represents the actual 6MWD, while the y-axis represents the predicted 6MWD. The black dots represent individual data points, and the red line represents the linear regression line fitted to the data.

Then the authors evaluated the prediction performance of the Random Forest model. The fitted plot after the Random Forest regression is shown in Fig. 5. The red line in the figure indicates a perfect fit. Most of the predicted values are observed to be scattered around the perfect line. The R^2^-value of 0.806 indicates a strong correlation between the predicted and actual values, suggesting that the Random Forest model effectively captures the relationship between digital 1MSTST and 6MWT.

**Fig. 6:**
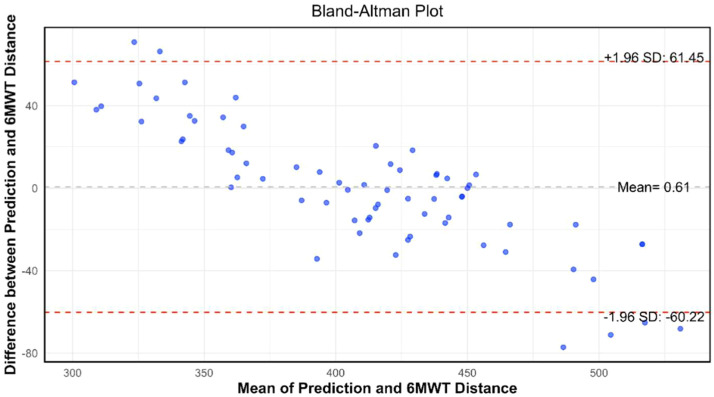
Bland-Altman analysis for the estimated 6MWD of the Random Forest model.

Bland-Altman analysis was conducted to assess the agreement between the 1MSTST algorithm's predicted 6MWD and the actual 6MWD (Fig. 6). The results indicate that the estimation model provides a bias of −0.61 ± 31.04 and that the limits of agreement (95%, 1.96 SD) range from −60.22 to 61.45. This observation suggests a satisfactory level of measurement consistency between the predicted and actual values, indicating that the 1MSTST algorithm exhibits acceptable agreement with the 6MWT in estimating 6MWD.

## Discussion

The present study established that a smartphone-based 1MSTST, analyzed via a random forest model, can accurately and simply predict the 6MWD in COPD patients. This conclusion was supported by physiological consistency, as the 1MSTST demonstrated a consistent pattern of ΔSpO_2_ with the 6MWT but provoked a more rapid heart rate increase. Critically, the authors distilled 39 IMU-derived features down to 7 highly explanatory parameters that capture the movement patterns during the 1MSTST and show a strong correlation with the 6MWD. Leveraging these key features, the authors developed and compared multiple machine learning models (Random Forest, Linear Regression, SVM, XGBoost, LightGBM). The Random Forest model was ultimately identified as the superior predictor, achieving the best fit (R^2^ = 0.806) and demonstrating strong agreement between the predicted and actual 6MWD values. To our knowledge, this is the first study to propose and apply a digital 1MSTST using a single smartphone to accurately predict 6MWD and objectively assess the exercise endurance impairment in patients with COPD.

> 300 million people worldwide suffer from COPD, and this large and growing global burden makes COPD a public health problem.^15^^,^^16^ The results of an epidemiological survey in China show that the number of patients with COPD is estimated to be nearly 100 million.^17^ More and more elderly COPD patients need long-term health monitoring. The 6MWT has emerged as a reliable measure of functional capacity, which can help capture extrapulmonary manifestations of COPD that typically co-exist, including cardiovascular disease, weakness, and sarcopenia.^18^^,^^19^ A shorter 6MWD was associated with increased mortality in 13 (93%) out of the 14 COPD studies reviewed.^2^ Nevertheless, a large meta-analysis showed a quasi-linear increase in the risk of death and hospitalization with 6MWD below a threshold of 350 m.^20^ However, the 6MWT received environmental and conditioning limitations that are not conducive to long-term home monitoring. A study has found that 1MSTST can predict the mortality rate of patients with COPD, and it can be easily implemented across practice settings.^21^ The 1MSTST has been proved to be a reliable, flexible and responsive alternative method, which showed comparable exercise physiological responses to the 6MWT.^5^^,^^22^

In a recent publication, the World Health Organization (WHO) outlined a digital health strategy that emphasizes the significance of patient engagement.^23^ The long-term monitoring of patients' physical function is feasible through the utilization of smart devices. Sensors have proven valuable for monitoring daily activity and function.^24^^,^^25^ The inertial sensors (e.g., accelerometers and gyroscopes) on smartphones can be used to capture the kinematic properties of human movement. 26, 27, 28 Further algorithmic advances may enable more nuanced interpretation of functional gains and declines over time and with interventions.^29^ These sensors can be used to develop machine learning models that offer a reliable, cost-effective, and wearable alternative for the assessment of postural transfers and mobility in patients with COPD.

What’s more, the authors built a smartphone application on the LucaPlex® platform and run on an iPhone 12. The launch of the mobile app effectively predicts 6MWD in a domestic setting, thereby suggesting cardiorespiratory fitness in patients with COPD. In the present study, no adverse events occurred during the 1MSTST, which proves that smartphone-based 1MSTST is safe and reliable.

The present study did not identify any statistically significant differences in the changes observed in SpO_2_ and DBP following the 1MSTST and 6MWT. However, HR and SBP demonstrated a more pronounced elevation following the 1MSTST. Compared with the 6MWT, 1MSTST was observed to induce a greater cardiorespiratory stress. This may be associated with the elevated requirements of 1MSTST on lower limb muscle strength and whole-body balance. Actually, the 1MSTST has been used as a surrogate index of muscle endurance.^30^^,^^31^

These data preliminarily identified seven highly correlated and interpretable parameters. Features like sit-stand counts, mean sit-stand time, and gravitational acceleration changes represent basic mobility, agility, and balance. Sample entropy assesses movement pattern complexity. Forward trunk angle indicates posture control and stability during sit-stands. The previous study reported larger maximum trunk angle during the sit-to-stand transition in patients diagnosed with hip or knee osteoarthritis compared to those without osteoarthritis.^5^ The percentage of sit-stand time reveals movement rhythm and timing precision. Collectively, these parameters provide insight into patients’ functional capacities to track progress and guide targeted interventions for improving mobility, stability, coordination, and movement rhythm.

As a single-center study with a small sample size, the potential for selection bias cannot be excluded. Future directions include optimizing the unsupervised digital 1MSTST model and validating it in a larger COPD cohort. The digital 1MSTST holds promise as a more accurate, reliable, and convenient digital biomarker compared to traditional 6MWT and 1MSTST assessments. This technique could be used to develop future telehealth solutions, including smartphone-based applications which have the potential to aid decision-making and self-monitoring in the management of COPD patients.

## Conclusions

In conclusion, these findings demonstrate that smartphone-based digital 1MSTST, analyzed with a Random Forest machine learning model, can accurately estimate 6MWD in patients with COPD. The findings suggest that digital 1MSTST can provide a safe, objective, and easily deployable alternative to 6MWT, facilitating continuous home-based monitoring and personalized disease management.

## Data availability

Restrictions apply to the availability of data generated or analyzed during this study to preserve patient confidentiality or because they were used under license. Data are, however, available from the authors upon reasonable request.

## Ethics approval and consent to participate

The Ethics Committee of Ruijin Hospital, Shanghai Jiao Tong University School of Medicine approved this study (Approval n° 2023–199). This study did not involve any animal or human specimen experiments. All participants provided informed consent before enrollment.

## Consent for publication

All the participants gave informed consent for the collection of data.

## Authors’ contributions

Jieming Qu, Min Zhou and Yi Guo conceived and designed the study. Simin Xie, Xiao Ge and Lin Huang collect the 1MSTS and 6MWT data from 66-patients. Wenyu Zhu and Yue Yang analyzed the data. Min Zhou funded this research. Simin Xie, Yue Yang and Xiao Ge drafted the manuscript. Jieming Qu, Min Zhou and Yi Guo revised the draft of the manuscript. All authors read and approved the final manuscript for publication.

## Funding

This work was supported by the National Key R&D Program of China [2022YFC2010005].

## Declaration of competing interest

The authors declare no conflicts of interest.

## References

[1] Lozano R., Naghavi M., Foreman K. (2012). Global and regional mortality from 235 causes of death for 20 age groups in 1990 and 2010: a systematic analysis for the Global Burden of Disease Study 2010. Lancet.

[2] Singh S.J., Puhan M.A., Andrianopoulos V. (2014). An official systematic review of the European respiratory society/American thoracic society: measurement properties of field walking tests in chronic respiratory disease. Eur Respir J.

[3] Holland A.E., Spruit M.A., Troosters T. (2014). An official European respiratory society/American thoracic society technical standard: field walking tests in chronic respiratory disease. Eur Respir J.

[4] Csuka M., McCarty D.J. (1985). Simple method for measurement of lower extremity muscle strength. Am J Med.

[5] Crook S., Büsching G., Schultz K. (2017). A multicentre validation of the 1-min sit-to-stand test in patients with COPD. Eur Respir J.

[6] Ozalevli S., Ozden A., Itil O. (2007). Comparison of the sit-to-stand test with 6 min walk test in patients with chronic obstructive pulmonary disease. Respir Med.

[7] Rausch-Osthoff A.K., Kohler M., Sievi N.A. (2014). Association between peripheral muscle strength, exercise performance, and physical activity in daily life in patients with Chronic obstructive pulmonary disease. Multidiscip Respir Med.

[8] Vaidya T., de Bisschop C., Beaumont M. (2016). Is the 1-minute sit-to-stand test a good tool for the evaluation of the impact of pulmonary rehabilitation? Determination of the minimal important difference in COPD. Int J Chron Obs Pulmon Dis.

[9] Puhan M.A., Siebeling L., Zoller M. (2013). Simple functional performance tests and mortality in COPD. Eur Respir J.

[10] Marques D.L., Neiva H.P., Pires I.M. (2021). An experimental study on the validity and reliability of a smartphone application to acquire temporal variables during the single sit-to-stand test with older adults. Sensors.

[11] Boswell M.A., Kidziński Ł., Hicks J.L. (2023). Smartphone videos of the sit-to-stand test predict osteoarthritis and health outcomes in a nationwide study. NPJ Digit Med.

[12] Agbohessou K.G., Sahuguede S., Lacroix J. (2023). Validity of estimated results from a wearable device for the tests time up and go and sit to stand in young adults and in people with chronic diseases. Sens (Basel).

[13] Watson K., Winship P., Cavalheri V. (2023). In adults with advanced lung disease, the 1-minute sit-to-stand test underestimates exertional desaturation compared with the 6-minute walk test: an observational study. J Physiother.

[14] Bohannon R.W., Crouch R. (2019). 1-Minute sit-to-stand test: systematic review of procedures, performance, and clinimetric properties. J Cardiopulm Rehabil Prev.

[15] Stolz D., Mkorombindo T., Schumann D.M. (2022). Towards the elimination of chronic obstructive pulmonary disease: a Lancet commission. Lancet.

[16] Safiri S., Carson-Chahhoud K., Noori M. (2022). Burden of chronic obstructive pulmonary disease and its attributable risk factors in 204 countries and territories, 1990-2019: results from the Global burden of disease study 2019. BMJ.

[17] Wang C., Xu J., Yang L. (2018). Prevalence and risk factors of chronic obstructive pulmonary disease in China (the China Pulmonary Health [CPH] study): a national cross-sectional study. Lancet.

[18] Celli B.R., Cote C.G., Lareau S.C. (2008). Predictors of survival in COPD: more than just the FEV1. Respir Med.

[19] Gale N.S., Albarrati A.M., Munnery M.M. (2018). Frailty: a global measure of the multisystem impact of COPD. Chron Respir Dis.

[20] Celli B., Tetzlaff K., Criner G. (2016). The 6-minute-walk distance test as a chronic obstructive pulmonary disease stratification tool. Insights from the COPD biomarker qualification consortium. Am J Respir Crit Care Med.

[21] Maddocks M., Nolan C.M., Man W.D. (2017). Simple functional tests in COPD: stand up and be counted!. Eur Respir J.

[22] Reychler G., Boucard E., Peran L. (2018). One minute sit-to-stand test is an alternative to 6MWT to measure functional exercise performance in COPD patients. Clin Respir J.

[23] World Health O (2021).

[24] Woelfle T., Bourguignon L., Lorscheider J. (2023). Wearable sensor technologies to assess motor functions in people with Multiple sclerosis: systematic scoping review and perspective. J Med Internet Res.

[25] Sapienza S., Tsurkalenko O., Giraitis M. (2024). Assessing the clinical utility of inertial sensors for home monitoring in Parkinson's disease: a comprehensive review. NPJ Parkinsons Dis.

[26] Lugade V., Fortune E., Morrow M. (2014). Validity of using tri-axial accelerometers to measure human movement - part I: posture and movement detection. Med Eng Phys.

[27] Millor N., Lecumberri P., Gomez M. (2014). Drift-free position estimation for periodic movements using inertial units. IEEE J Biomed Health Inf.

[28] Hayek R., Gutman I., Baranes G. (2024). Smartphone-based sit-to-stand analysis for mobility assessment in middle age. Innov Aging.

[29] Wairagkar M., Villeneuve E., King R. (2022). A novel approach for modelling and classifying sit-to-stand kinematics using inertial sensors. PLoS One.

[30] Ritchie C., Trost S.G., Brown W. (2005). Reliability and validity of physical fitness field tests for adults aged 55 to 70 years. J Sci Med Sport.

[31] Segura-Ortí E., Rodilla-Alama V., Lisón J.F. (2008). Physiotherapy during hemodialysis: results of a progressive resistance-training programme. Nefrologia.

